# Indigenous Lands, Protected Areas, and Slowing Climate Change

**DOI:** 10.1371/journal.pbio.1000331

**Published:** 2010-03-16

**Authors:** Taylor H. Ricketts, Britaldo Soares-Filho, Gustavo A. B. da Fonseca, Daniel Nepstad, Alexander Pfaff, Annie Petsonk, Anthony Anderson, Doug Boucher, Andrea Cattaneo, Marc Conte, Ken Creighton, Lawrence Linden, Claudio Maretti, Paulo Moutinho, Roger Ullman, Ray Victurine

**Affiliations:** 1 World Wildlife Fund, Washington, D.C., United States of America; 2 Universidade Federal de Minas Gerais, Belo Horizonte, Brazil; 3 Global Environment Facility, Washington, D.C., United States of America; 4 Woods Hole Research Center, Falmouth, Massachusetts, United States of America; 5 Instituto de Pesquisa Ambiental da Amazonia, Belem, Brazil; 6 Duke University, Durham, North Carolina, United States of America; 7 Environmental Defense Fund, Washington, D.C., United States of America; 8 World Wildlife Fund-Brazil, Brasilia, Brazil; 9 Union of Concerned Scientists, Washington, D.C., United States of America; 10 Stanford University, Stanford, California, United States of America; 11 WWF-Belgium, Brussels, Belgium; 12 Linden Trust for Conservation, New York, New York, United States of America; 13 Wildlife Conservation Society, Bronx, New York, United, States of America

## Abstract

Recent climate talks in Copenhagen reaffirmed the crucial role of reducing emissions from deforestation and degradation (REDD). Creating and strengthening indigenous lands and other protected areas represents an effective, practical, and immediate REDD strategy that addresses both biodiversity and climate crises at once.

Forest clearing and degradation account for roughly 15% of global greenhouse gas emissions, more than all the cars, trains, planes, ships, and trucks on earth [1],[2]. This is simply too big a piece of the problem to ignore; fail to reduce it and we will fail to stabilize our climate [3].

Although the recent climate summit in Copenhagen failed to produce a legally binding treaty, the importance of forest conservation in mitigating climate change was a rare point of agreement between developed and developing countries and is emphasized in the resulting Copenhagen Accord [4],[5]. Language from the meeting calls for developing countries to reduce emissions from deforestation and degradation (nicknamed REDD), and for wealthy nations to compensate them for doing so [4],[6]–[8].

For REDD to succeed, forest nations must develop policies and institutions to reduce and eventually eliminate forest clearing and degradation [9]. One of the most straightforward components of such a program is also one of the oldest and most reliable tricks in the conservation book: protected areas. Indigenous lands and other protected areas (hereafter ILPAs [10]–[12])—created to safeguard land rights, indigenous livelihoods, biodiversity, and other values—contain more than 312 billion tons of carbon (GtC) [13]. Crucially, and paradoxically, this “protected carbon” is not entirely protected. While ILPAs typically reduce rates of deforestation compared to surrounding areas [14]–[18], deforestation (with resulting greenhouse gas [GHG] emissions) often continues within them, especially inside those that lack sufficient funding, management capacity, or political backing [19].

These facts suggest an attractive but overlooked opportunity to reduce GHG emissions: creating new ILPAs and strengthening existing ones [20]. Here, we evaluate the case for this potential REDD strategy. We focus on the Amazon basin given its importance for global biodiversity, its enormous carbon stocks, and its advanced network of indigenous lands and other protected areas [16],[21].

## The Policy Playing Field

Several policy alternatives for REDD have been under negotiation, both in Copenhagen and elsewhere. One approach is for developed nations to capitalize funds to reduce GHG emissions in developing countries. For example, the Amazon Fund, initially capitalized by Norway, will help to finance REDD efforts in the Brazilian Amazon [9],[22]. A second approach is compliance markets, in which nations or regulated entities must reduce their emissions or buy offsets from others. This approach will take more time, but negotiations are under way to develop or expand compliance markets for REDD within the United Nations Framework Convention on Climate Change (UNFCCC), the European Union, and the United States [6],[8],[9],[23].

Both of these frameworks—for the near term, at least—will likely emphasize reductions in carbon emissions compared against national baselines [6],[7],[24]. This crucial point has two implications here. First, although Brazil's Amazonian forests contain 47+-9 GtC [9], Brazil will be primarily compensated not for these stocks, but for slowing the net rate of loss from them (i.e., reducing carbon emissions). Second, countries will estimate their nationwide emissions baselines and then earn international compensation for reductions below this baseline [7]. It will be up to each nation to decide how to achieve these reductions (e.g., protecting forests, redirecting drivers of deforestation, and other land-based strategies), and how to allocate any payments received.

## The Role for ILPAs

Given this likely policy landscape, nations can use ILPAs to reduce emissions in two ways: first, create new ILPAs in areas facing deforestation risk now and in the foreseeable future; second, strengthen the management of existing ILPAs to reduce ongoing deforestation within and surrounding their borders.

**Figure 1:**
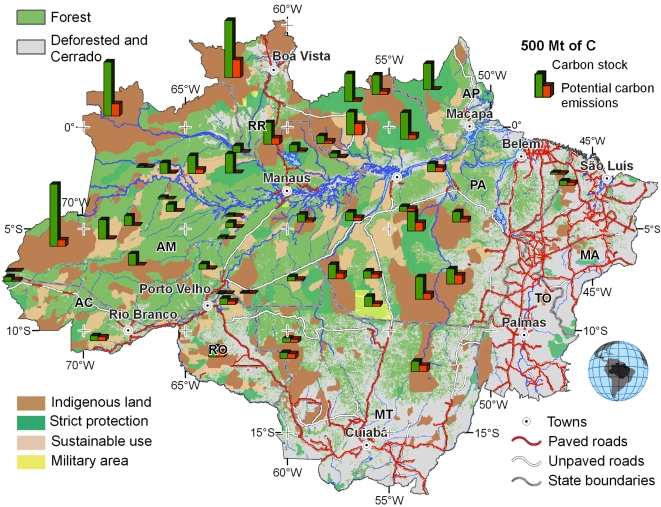
Carbon stocks and potential emissions of selected ILPAs in the Brazilian Amazon. Potential emissions are estimated by simulating future deforestation through 2050, with and without ILPAs present. The difference (depicted by orange bars) represents the reductions of CO_2_ emissions contributed by each ILPA. Figure and data modified from Soares and colleagues [15],[16].

That new ILPAs reduce deforestation may seem an obvious point, but how much? Since 2002 in the Brazilian Amazon, the average probability of deforestation has been 7–11 times lower inside ILPAs than in surrounding areas. Simulation models suggest that ILPAs established between 2003 and 2007 could prevent 272,000 km^2^ of deforestation through 2050, equal to 3.3 +-1.1 GtC, more than 1/3 of the world's annual CO_2_e emissions (Figure 1) [15]. Bolivia's Noel Kempff Mercado National Park, which expanded by 8,317 km^2^ in 1997, is projected to prevent emission of up to 1.6 million tC over 30 years [25].

Less obvious is that despite impressive success in these and other cases, ILPAs do not reduce deforestation risk to zero. Protected sites in the Brazilian Amazon lost 9,700 km^2^ of forest cover between 2002 and 2007, representing 8% of Amazon deforestation within this time period [15]. Improving the protection of *existing* ILPAs can therefore reduce emissions even further.

To be meaningful components of a national REDD strategy, ILPAs must reduce GHG emissions below what would have happened had they never been established. Careful analysis of this counterfactual can reveal surprising and often controversial results. For example, although Brazil's Chico Mendes Extractive Reserve continues to suffer deforestation, without the reserve an additional 7% of the area would have been lost in each of the last two decades [26]. By comparison, other nearby reserves (e.g., Chandless State Park), farther from the pressures associated with the Interoceanic Highway, are hardly deforested but would be little different without protection. So which is the more effective contributor to REDD? Rigorous analyses point to Chico Mendes [26]. In general, carefully assessing impact and counterfactuals will allow nations to focus REDD resources where meaningful reductions are most likely and to design national programs that, in effect, give credit where credit is due.

Guided by such analyses, national REDD programs may tend to focus investments on areas under high development pressure (e.g., along the BR-163 Cuiabá–Santarém highway or within the southeastern Amazon's agricultural frontier). On one hand, these areas are exactly where enhanced funds may be most needed, to bolster enforcement and cover higher opportunity costs [9]. On the other hand, this may shift resources away from highly biodiverse but remote regions [27]. With human population and forest threats continuing to expand [28], even wildernesses face some non-zero future threat (Figure 1). Further, the introduction of REDD payments focused on high-pressure areas could displace deforestation to remote areas (i.e., cause “leakage” of emissions). These and other concerns have led negotiators to propose “REDD-plus,” in which credits would be awarded not only for reducing deforestation and degradation, but also for conserving forest carbon stocks and managing forests sustainably [6]. This proposal could reduce leakage by rewarding conservation of high-carbon, low-threat forests and could improve buy-in by compensating different REDD activities, locations, and stakeholders.

Eventually, funding from developed nations could enable national and subnational governments to implement comprehensive REDD programs with formal overall targets [6]. Brazil, for example, has recently taken on such targets (e.g., reducing Amazon deforestation 80%; [29]), as have four Brazilian Amazon states. Success in these programs will hinge on meeting their overall targets, allowing nations to invest in ILPAs without knowing the exact contributions of each one. While this would reduce costs of carefully evaluating deforestation risk for each ILPA, rigorous analysis of impacts and counterfactuals would still help optimally direct funds within a national REDD program [9].

How much would creating and better protecting existing ILPAs cost? Completing and managing a network of protected areas in developing countries would require an estimated US$4 billion per year (up from < US$1 billion currently spent annually) [30]. This represents only 9–13% of the capital that could be mobilized by international REDD frameworks at a price of US$5/tonCO_2_e [31]. For the Brazilian Amazon, Nepstad et al. [9] estimate that REDD will cost US$1–2/tonCO_2_e, including payments to forest peoples programs, partial compensation of opportunity costs, enhanced law enforcement, and greater funding for ILPAs. These costs are far lower than those estimated for many other options to reduce emissions [32].

ILPAs may be more cost effective than other REDD strategies, in part because they would be more straightforward to implement. First, the act of declaring an ILPA typically clarifies land tenure and associated carbon rights (provided appropriate safeguards have been met, particularly related to indigenous peoples). Second, ILPAs are “ready to go.” Protected areas departments, indigenous peoples agencies, and related institutions often already exist with budgets and staff and infrastructure to receive REDD payments, strengthen protection, and generate results quickly (e.g., Brazil's ARPA program [15]). Third, directing REDD funds appropriately can be straightforward. ILPAs are typically funded by governments, so payments can simply take the form of increased funding. In contrast, distributing payments to thousands of private landowners in a fair and transparent way will be more difficult (but not impossible; see examples in Costa Rica [33] and elsewhere, and a proposal for the Brazilian Amazon [9]).

Crucially, ILPAs offer multiple benefits beyond emissions reductions. They protect biodiversity and indigenous land rights, as they are designed to do. Furthermore, they can purify water, provide food to local communities, regulate regional climate, and maintain culturally important elements of the landscape [34].

## Taking Action

So what can national governments do to include ILPAs effectively in their REDD strategies? One obvious step is to identify where establishing or strengthening ILPAs would most effectively reduce emissions (Figure 1). The studies discussed here show that spatial data and techniques exist to estimate effectiveness rigorously [15],[16],[18],[25],[26]. A second and urgent step is to establish national monitoring schemes to measure deforestation rates and quantify carbon emissions reductions. Brazil's system of remotely sensed monitoring and Noel Kempff's network of on-the-ground monitoring plots are good models [25],[35]. A third step is to establish insurance mechanisms, pooling the risk that illegal logging or fires reverse gains in individual ILPAs.

Finally, governments must provide indigenous groups and local communities the information and capacities they need to participate, and payments must be distributed transparently to reward those responsible for reducing emissions. In Brazil, indigenous lands currently contribute far more to REDD than parks or nature reserves because they cover three times the area and are often in the immediate path of the expanding agricultural frontier [17]. The science community can support nations in all of these efforts by illuminating several simple questions with nuanced answers (see Box 1).

**Box 1. What science is needed?**

To include ILPAs effectively in REDD strategies, nations will need answers to several critical science questions, including:

- How effective are ILPAs in reducing forest emissions? Rigorous estimates of emissions reduced by ILPAs are feasible [15],[16],[18],[25],[26], will increase credibility of national REDD programs, and will help provide technical basis for in-country allocation of funds.
- Where should ILPA investments be targeted? Maps of carbon stocks, deforestation risk, and opportunity costs would allow nations to assess where investments in ILPAs would reduce most emissions at least cost. Formal optimization algorithms [36] could be used to prioritize action.
- Do better funded ILPAs emit less carbon? REDD funds can strengthen existing ILPAs and reduce deforestation inside their borders, but this relationship needs to be examined empirically. Are there diminishing returns to additional funds? Thresholds? Specific guidelines can help protected areas system managers target limited resources.
- How does the governance of ILPAs—in particular recognition of indigenous land rights and local control—impact their effectiveness in reducing emissions? There is increasing evidence that local ownership over forest commons improves both carbon storage and local livelihoods (e.g., [37]). Ensuring good governance may therefore improve the effectiveness of funds steered toward ILPAs to reduce forest clearing and degradation.
- What about the second “D”, forest degradation? Asner et al. [38] estimate that as much as 20% of forest emissions in the Brazilian Amazon are due to selective logging and associated forest degradation. But almost all research and monitoring has focused on deforestation per se. How effective are ILPAs in reducing this under-studied component of REDD? How does that depend on their location, their funding levels, and the causes of degradation?

ILPAs are only one part of national REDD programs, and REDD is only one of many mechanisms to reduce land-based emissions. Nevertheless, REDD is likely to be the first such mechanism to take international effect, and ILPAs clearly can make an early and important contribution. The world therefore faces an unprecedented opportunity to address two problems at once: mitigating climate change while securing our planet's vital natural and cultural heritage. Win-wins don't get better than that.

## Abbreviations

ARPA: Amazon Region Protected Areas
GHG: Greenhouse Gas
ILPAs: Indigenous Lands and Protected Areas
REDD: Reduced Emissions from Deforestation and Degradation
UNFCCC: United Nations Framework Convention on Climate Change
